# The MEPS server for identifying protein conformational epitopes

**DOI:** 10.1186/1471-2105-8-S1-S6

**Published:** 2007-03-08

**Authors:** Tiziana Castrignanò, Paolo D'Onorio De Meo, Danilo Carrabino, Massimilano Orsini, Matteo Floris, Anna Tramontano

**Affiliations:** 1 CASPUR, Consorzio Interuniversitario per le Applicazioni di Supercalcolo per Universita' e Ricerca, Via dei Tizii, 6/b, 5 I-00185 Rome, Italy; 2 Center for Advanced Studies, Research and Development in Sardinia (CRS4), Bioinformatics Unit, Parco Scientifico e Tecnologico, POLARIS, Edificio 1, 09010 PULA (CA), Italy; 3 Department of Biochemical Sciences, University 'La Sapienza', P.le Aldo Moro, 5, I-00185 Rome, Italy; 4 Istituto Pasteur – Fondazione Cenci Bolognetti, University 'La Sapienza', P.le Aldo Moro, 5, I-00185 Rome, Italy

## Abstract

**Background:**

One of the most interesting problems in molecular immunology is epitope mapping, i.e. the identification of the regions of interaction between an antigen and an antibody. The solution to this problem, even if approximate, would help in designing experiments to precisely map the residues involved in the interaction and could be instrumental both in designing peptides able to mimic the interacting surface of the antigen and in understanding where immunologically important regions are located in its three-dimensional structure. From an experimental point of view, both genetically encoded and chemically synthesised peptide libraries can be used to identify sequences recognized by a given antibody. The problem then arises of which region of a folded protein the selected peptides correspond to.

**Results:**

We have developed a method able to find the surface region of a protein that can be effectively mimicked by a peptide, given the structure of the protein and the maximum number of side chains deemed to be required for recognition. The method is implemented as a publicly available server. It can also find and report all peptide sequences of a specified length that can mimic the surface of a given protein and store them in a database.

The immediate application of the server is the mapping of antibody epitopes, however the system is sufficiently flexible for allowing other questions to be asked, for example one can compare the peptides representing the surface of two proteins known to interact with the same macromolecule to find which is the most likely interacting region.

**Conclusion:**

We believe that the MEPS server, available at , will be a useful tool for immunologists and structural and computational biologists. We plan to use it ourselves to implement a database of "surface mimicking peptides" for all proteins of known structure and proteins that can be reliably modelled by comparative modelling.

## Background

Antibodies recognize their cognate antigen by establishing energetically favourable interaction with its amino acids [1]. In some cases, the recognition site is represented by a continuous segment of the antigen sequence, but much more often the epitope is "conformational", i.e. the antibody recognizes the location and type of exposed antigen side chains that are not necessarily contiguous in the antigen's sequence, but brought together by its three-dimensional structure [2]. Identifying the location of the epitope on the protein, i.e. mapping it, is of paramount importance because it is instrumental for the development of diagnostic or biotechnological tools and of recombinant vaccines [3-7].

Epitope mapping can be achieved by experimental techniques. If the target protein is known and cloned, one can fragment or sub-clone the protein and identify which region is still recognized by the antibody [3]. Clearly only continuous epitopes, or epitopes that contain at least one reasonably long stretch of contiguous target amino acids, can be identified with this strategy.

The correct identification of a conformational epitope can be obtained by structural determination of the antibody antigen complex, a time and labour consuming procedure that cannot be guaranteed to succeed in all cases.

However, molecular biology and peptide chemistry provide two powerful tools for mapping the epitopes of a protein, given the recognizing antibody: combinatorial libraries.

Both genetically encoded and chemically synthesised peptide libraries have been used with great success in this area [8,9]. The great diversity contained in a library allows the selection of peptides able to bind to an antibody. The analysis of their sequences can allow the location of linear epitopes to be immediately identified by just comparing the sequences of the peptides with that of the antigen [10].

It is worth emphasizing that the library selection strategy does not require the knowledge of the antigen, but only the availability of an antibody, thereby it can also be used for identifying unknown agents provided antibodies against them can be elicited or found. Furthermore, although antibodies are the most commonly used selectors in combinatorial library experiments, because they can be obtained from patient sera or by immunizing experimental animals, it should be apparent that the technique can be, and is, applied using any receptor as selector molecule.

The experimental strategy does not necessarily limit the results to peptides mimicking a continuous epitope, since peptides able to mimic the discontinuous surface of a conformational epitope can be selected as well. In these cases, though, a straightforward sequence comparison is not sufficient to identify the original epitope on the target protein even more so if the task is not only the identification of the epitope on a given protein, but also the identification of the recognized protein.

The problem in this case is to find a surface region of a protein that can be effectively mimicked by one or more selected peptides, given the structure of the protein and the maximum number of side chains deemed to be required for recognition.

A method for solving the problem has been previously described by some of us [11], but at the time technology and computer power was not adequate to implement the method as a publicly available server and for extending its use to a large number of proteins, and therefore it could only be used locally for the detection of epitopes of a known antigen and it was not suitable for the identification of unknown antigens.

The MEPS server, described below, has been designed to fulfil these requirements.

## Results

### Generating the surface ensemble

We define here a surface ensemble as the collection of all peptides of a given length L that can position their side chains in such a way that at least m (1 < m < L) of their side chains are able to mimic exposed regions of the protein surface.

Given the structure of a target protein, we first select all solvent exposed amino acids. In the current implementation the threshold for minimum solvent accessible surface is set to 40 Å^2^. Next, we compute the distance between the Cβ (Cα for glycines) of each pair of exposed amino acids and store them in a matrix. The choice of using Cβ derives from the need of taking into account the direction of the side chain.

The matrix is used to build a graph where each node represents a surface amino acid, and an edge connects two nodes if their distance is lower than a maximum distance threshold *d*. The graph is represented as a collection of adjacency lists: there is a list for each amino acid and each list contains a pair [*neighbour*, *neigh_distance*] if *neigh_distance *is lower than *d*.

### Retrieving all putative epitopes for a target or a set of target proteins

The peptide ensemble graph is processed to extract the sequences of all possible peptides able to mimic the surface of a given protein, or of a set of protein structures. For each adjacency list, we search all neighbours within a 5.5 Å distance. The search is recursively repeated for each of the identified neighbours until its length reaches the value *l *selected by the user. The peptide sequences can be interactively inspected and/or stored in a file in FASTA format. This allows sequence similarity searching tools such as BLAST or FASTA to be directly used on the surface ensemble peptides.

The threshold is only applied to the distance between adjacent amino acids and therefore we implicitly list all peptides shorter than the maximum length selected by the user.

### Searching for a conformational epitope

The user can input a protein structure (or a set of protein structures), a peptide sequence and a minimum number of mismatching residues and retrieve the location, if it exists, of the corresponding epitope.

In this case, the system first exhaustively lists all possible patterns with mismatches (Table 1). Each pattern is searched in the graph, similarly to what is done for finding exact matches. In this case, however, the distance range between two adjacent amino acids depends upon the number of intervening mismatches.

The minimum and maximum distances between Cβ (Cα for glycines) of amino acids separated by 1, 2, 3, etc amino acids have been obtained by a statistical analysis of well resolved structures in the protein structure databank (PDB) [12], consequently they are not only chemically possible, but also stereochemically plausible.

### User interface

**Figure 1:**
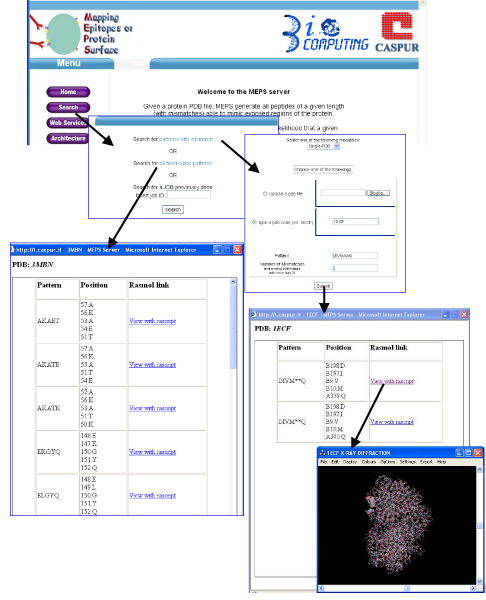
**Using MEPS**. The Figure shows a search for a pattern with mismatches (DIVMAAQ) in the 1ECF protein structure and the results of a search for all fixed size patterns of length 5 able to mimic the surface of the 3MBN protein structure.

Figure 1 shows the entry page of the MEPS server and the page displayed upon selecting the "Search" button. The user can select the search mode, or retrieve the result of a previously run job.

If the "pattern with mismatch" option is selected, several possibilities are offered: the user can upload a local PDB file, a file from the PDB database, or a set of PDB files.

In all cases, the user is asked for the searched pattern and the maximum number of mismatches. In the example shown, the user has selected the 1ECF protein structure and the pattern "DIVMAAQ" allowing two mismatches. The protein contains two overlapping sets of residues that can mimic the input peptide (the last glutamine can mimic residue 339 or 340). The user can visualize the location of the peptide on the protein structure via Rasmol, by clicking on the appropriate link.

If the "all fixed-size patterns" option is selected, the user only needs to select the target protein(s) and choose the length of the peptides. In the case shown in the figure, the 3MBN entry of the PDB database is selected. The list of peptides (length 5 in the example) is shown in a new window, together with the rasmol links. They can also be stored as a fasta file that can be used for further analyses. The header of each of the sequences in the fasta file lists the position in the structure of the residues of the peptides.

## Discussion

The MEPS server has been designed to answer the question of which regions of a known or modelled protein structure can be mimicked by a peptide.

Although our main interest in developing the server was to enable user to map antibody epitopes, it is clear that the method can be used for many other purposes. For example, for designing peptides able to mimic a portion of two interacting proteins, or for identifying common surface patches among two or more proteins.

## Conclusion

New functionalities to the basic structure of the server described are already being implemented. These include the possibility of selecting the target proteins on the basis of a keyword search and of comparing the surface ensemble of two different proteins.

## Methods

### Implementation details

The MEPS web server is freely available [13]. It is implemented on a 4-processor Linux server (HP DL585 with SUSE SLESS 9) running the apache web server version 2.0 [14] and has been developed as a Service-Oriented Architecture (SOA).

The MEPS interface developed in PHP [15] allows the user to submit one or more protein structures in PDB format. Upon submission, PHP scripts, running in background, call three web services through the Simple Object Access Protocol (SOAP) open standard. When the job is complete, the PHP interface shows a table of results. Each pattern can be displayed in the context of the protein structure using a standalone visualization tool (for windows and linux systems) based on the 32 bit version of the open source protein visualization tool Rasmol^© ^(1994 Roger Sayle) (available through the server). WSs have been implemented in java enterprise 1.5; all WSs details are described in [13].

### Distance thresholds

The minimum and maximum distances between Cβ (Cα for glycines) of amino acids separated by 1, 2, 3, etc amino acids have been obtained by a statistical analysis of well resolved structures in the protein structure databank (PDB). The computation was performed using a script based on BioPython classes. The data set included single chain protein structures in PDB with a resolution of 2.0 Å or better.

## Authors' contributions

PDD and DC contributed to writing the computer code. MO and MF analyzed the PDB data base entries for deriving parameters and extensively tested the server. TC and AT developed the methodology. All authors contributed to writing the manuscript.

## Figures and Tables

**Table 1:** Searching pattern with mismatches. Example of patterns with up to three mismatches for the input peptide AYKTK

mismatches	1	2	3
Patterns	*YKTK	**KTK	***TK
	A*KTK	*Y*TK	**K*K
	AY*TK	*YK*K	**KT*
	AYK*K	*YKT*	*Y**K
	AYKT*	A**TK	*Y*T*
		A*K*K	*YK**
		A*KT*	A***K
		AY**K	A**T*
		AY*T*	A*K**
		AYK**	AY***
